# Phenotypic drug-susceptibility profiles and genetic analysis based on whole-genome sequencing of *Mycobacterium avium* complex isolates in Thailand

**DOI:** 10.1371/journal.pone.0294677

**Published:** 2023-11-22

**Authors:** Auttawit Sirichoat, Orawee Kaewprasert, Yothin Hinwan, Kiatichai Faksri

**Affiliations:** 1 Department of Microbiology, Faculty of Medicine, Khon Kaen University, Khon Kaen, Thailand; 2 Research and Diagnostic Center for Emerging Infectious Diseases (RCEID), Khon Kaen University, Khon Kaen, Thailand; Showa University Fujigaoka Hospital, JAPAN

## Abstract

*Mycobacterium avium* complex (MAC) infections are a significant clinical challenge. Determining drug-susceptibility profiles and the genetic basis of drug resistance is crucial for guiding effective treatment strategies. This study aimed to determine the drug-susceptibility profiles of MAC clinical isolates and to investigate the genetic basis conferring drug resistance using whole-genome sequencing (WGS) analysis. Drug-susceptibility profiles based on minimum inhibitory concentration (MIC) assays were determined for 38 MAC clinical isolates (12 *Mycobacterium avium* and 26 *Mycobacterium intracellulare*). Mutations associated with drug resistance were identified through genome analysis of these isolates, and their phylogenetic relationships were also examined. Drug resistance, based on MIC values, was most commonly observed for moxifloxacin (81.6%), followed by linezolid (78.9%), clarithromycin (44.7%) and amikacin (36.8%). We identified specific mutations associated with resistance to amikacin. These include the *rrs* mutation at C464T in amikacin intermediate-resistance *M*. *avium*, and two mutations at T250A and G1453T in amikacin non-susceptible *M*. *intracellulare*. Mutations in *rrl* at A2058G, A2059C and A2059G were potentially linked to clarithromycin resistance. MAC clinical isolates not susceptible to linezolid exhibited mutations in *rplC* at G237C and C459T, as well as two *rplD* mutations at G443A and A489G. GyrB substitution Thr521Ala (T521A) was identified in moxifloxacin non-susceptible isolates, which may contribute to this resistance. A phylogeny of our MAC isolates revealed high levels of genetic diversity. Our findings suggest that the standard treatment regimen for MAC infections using moxifloxacin, linezolid, clarithromycin and amikacin may be driving development of resistance, potentially due to specific mutations. The combination of phenotypic and genotypic susceptibility testing can be valuable in guiding the clinical use of drugs for the treatment of MAC infections.

## Introduction

Nontuberculous mycobacteria (NTM) are ubiquitous organisms that can cause chronic disease which is increasing in incidence and prevalence globally [1]. Among NTM species, the *Mycobacterium avium* complex (MAC), which mainly consists of *Mycobacterium avium* and *Mycobacterium intracellulare*, are common pathogens found in patients [2] and in various natural environments [3]. MAC can cause a range of conditions, including pulmonary disease, skin and soft-tissue infections and disseminated infections [4]. Due to their diverse clinical manifestations and chronic nature, MAC infections are a significant concern for clinicians in terms of diagnosis and treatment.

*Mycobacterium avium* complex is difficult to treat. Treatment failure can be observed in approximately one-third of cases [5]. The extensive use of drugs in the treatment of MAC has led to an increase in drug-resistant isolates, which poses a significant public-health challenge [6, 7]. Drug-susceptibility testing (DST) is crucial for the effective management of MAC infections. Although the Clinical and Laboratory Standards Institute (CLSI) has established guidelines for DST in NTM, DST data for MAC are limited. Currently, there are recommended clinical breakpoints for four drugs (amikacin, clarithromycin, linezolid and moxifloxacin), but there is no standard interpretation of DSTs for many potentially appropriate drugs [8]. Also, there can be disagreement between results of *in vitro* (phenotypic) drug-susceptibility tests and observed *in vivo* resistance of NTM [9, 10]. The acquisition of resistance occurs via specific mutations or the horizontal transfer of antimicrobial resistance (AMR) genes. Previous studies have identified several mutations that confer drug resistance in MAC. For instance, mutations in the *rrs* gene confer aminoglycoside resistance [11]. Mutations in the *rrl* confer macrolide resistance [12], those in *gyrA* or *gyrB* genes confer fluoroquinolone resistance [13] and changes in *rplC* or *rplD* genes confer linezolid resistance [14]. Therefore, both phenotypic and genotypic DST can be used to guide the treatment options for MAC infections.

Recent advances in whole-genome sequencing (WGS) and bioinformatics have provided powerful tools for analyzing genetics in various microorganisms [15]. This technology yields vast amounts of information, including species identification, molecular epidemiology, and detection of virulence factors and AMR genes [16]. The high resolution of WGS data enables the identification of various mutations that confer drug resistance and the integration of WGS data with clinical metadata can provide insights into the mechanism of drug resistance [17]. In addition, WGS has revolutionized our understanding of the evolutionary relationships among microorganisms and their hosts, shedding light on the emergence and spread of infectious diseases [18].

Several studies have been conducted on drug resistance in MAC, including *M*. *avium*, *M*. *intracellulare* and other species. However, few have reported relevant drug-susceptibility profiles based on MIC tests and genome analysis. Therefore, we aimed to determine the drug-susceptibility profiles of 38 MAC clinical isolates from Thai patients and investigated the genetic basis that confers drug resistance using WGS analysis.

## Materials and methods

### MAC clinical isolates

In total, 38 MAC isolates, identified as *M*. *avium* or *M*. *intracellulare*, were randomly selected and included in this study. The isolates were identified using the INNO-LiPA MYCOBACTERIA v2 line-probe assay (LPA, Hain Lifesciences, Nehren, Germany) as recommended by the manufacturer [19]. These isolates were collected from NTM-infected patients through various clinical samples, such as sputum, tracheal suction, skin, pus, synovial fluid and other tissues between 2012 and 2016, and had been maintained as stock cultures at the Clinical Microbiology Laboratory at Srinagarind Hospital, Khon Kaen University, Khon Kaen, Thailand. NTM infection was diagnosed based on guidelines published by the American Thoracic Society and the Infectious Diseases Society of America (ATS/IDSA) [20]. All clinical isolates were sub-cultured on Lowenstein-Jensen media and incubated at 37°C for 7–14 days prior to drug-susceptibility testing.

The study protocol was approved by the Institutional Review Board (IRB) of Khon Kaen University Ethics Committee in Human Research (No. HE591454). Informed consent was waived for the use of medical data since patient information was anonymized and de-identified before analysis.

### Drug-susceptibility testing

The minimum inhibitory concentration (MIC) of each drug was determined using the Sensititre Slow Growing Myco SLOMYCOI assay (TREK Diagnostic Systems, West Sussex, UK) as recommended by the manufacturer and following the guidelines of the CLSI [8]. The plate contained serial 2-fold dilutions of 13 lyophilized drugs, including clarithromycin (CLA 0.06–64 μg/mL), amikacin (AMI 1–64 μg/mL), rifabutin (RFB 0.25–8 μg/mL), rifampicin (RIF 0.12–8 μg/mL), ethambutol (EMB 0.5–16 μg/mL), trimethoprim/sulfamethoxazole (SXT 0.12/2.38–8/152 μg/mL), ciprofloxacin (CIP 0.12–16 μg/mL), moxifloxacin (MXF 0.12–8 μg/mL), ethionamide (ETH 0.3–20 μg/mL), isoniazid (INH 0.25–8 μg/mL), doxycycline (DOX 0.12–16 μg/mL), linezolid (LZD 1–64 μg/mL) and streptomycin (STR 0.5–64 μg/mL). Briefly, individual MAC colonies were suspended in Sensititre Sterile Water (TREK Diagnostic Systems) and the turbidity of the supernatant was adjusted to 0.5 McFarland standard. Then, 50 μL of this suspension was added into Sensititre Mueller-Hinton broth (MHB) with oleic acid, albumin, dextrose and catalase (OADC) (TREK Diagnostic Systems), and 100 μL of this inoculum was added to each well of the SLOMYCOI plate. The plate was covered with a plastic seal and incubated at 37°C. The plate was read after 7 days of incubation, and in case of insufficient growth, the plate was re-incubated and read again after 10 to 14 days depending on the growth of mycobacteria in drug-free control wells. All processes were performed according to the standard operating procedure. The MIC was defined as the lowest concentration of drug that inhibited the growth of the tested isolate. The results were interpreted according to the CLSI guidelines [8].

For clarithromycin, amikacin, moxifloxacin, and linezolid, CLSI breakpoints [8] have been used to interpret MIC values (CLA: susceptible (S) ≤ 8 μg/mL, intermediate (I) = 16 μg/mL, resistant (R) ≥ 32 μg/mL; AMI: S ≤ 16 μg/mL, I = 32 μg/mL, R ≥ 64 μg/mL, MXF: S ≤ 1 μg/mL, I = 2 μg/mL, R ≥ 4, μg/mL, LZD: S ≤ 8 μg/mL, I = 16 μg/mL, R ≥ 32 μg/mL).

### Whole-genome sequencing

Genomic DNA was extracted from multiple loopfuls of MAC colonies using the cetyl-trimethylammonium bromide sodium chloride (CTAB) method [21]. The quality and concentration of the extracted genomic DNA were determined using the NanoDrop One (Thermo Fisher Scientific, Carlsbad, CA, USA), and the DNA was subsequently sent to a sequencing service company (NovogeneAIT in Hong Kong) for genome sequencing. A genomic library was constructed from the total genomic DNA of each of the 38 MAC isolates and was then subjected to paired-end sequencing on an Illumina HiSeq sequencer, generating 150-bp read lengths. The sequence data have been deposited in the Sequence Read Archive (SRA) under BioProject accession No. PRJNA972846.

### Bioinformatics and data analysis

#### Quality check and pre-processing

The quality of sequence reads was assessed using FastQC version 0.11.9 [22]. The average sequencing depth coverage was determined to be 147.5 (±10.7 standard deviations). All sequence reads longer than 75 bp were retained, while reads shorter than 75 bp and adapter sequences that could potentially contaminate the data were removed using Trimmomatic version 0.39 [23] with the options LEADING:3 TRAILING:3 SLIDINGWINDOW:4:15 MINLEN:75. The filtered reads were then used for downstream analysis.

#### Genome assembly and annotation

Paired-end filtered reads from each isolate were assembled using Unicycler version 0.4.8 [24] with default parameters. The quality of the resulting contigs was assessed using QUAST version 5.0.2 [25]. Gene prediction and functional annotation were carried out using the RAST tool kit (RASTtk) with BV-BRC web resources [26].

#### Phylogenetic analysis

Paired-end filtered reads of each isolate were mapped to the *Mycobacterium avium* 104 reference genome (GenBank accession number: CP000479.1) using BWA-MEM version 0.7.12 [27]. The mapped sequences were converted to SAM format then converted to BAM format, sorted, and indexed using SAMtools version 0.1.19 [28]. GATK version 3.4.0 [29] was used for local realignment of the mapped reads, and variant calling and filtering (including small indels as well as SNPs) was done using the intersection set of variants called by SAMtools and GATK. The variants were filtered based on a minimum coverage depth of 10X for each variant, a Q20 minimum base quality score and a C50 minimum mapping quality score. Heterozygous SNPs with allelic frequencies of less than 75% or read depths of less than 10 reads were excluded. SNPs that remained and satisfied all the above criteria were regarded as high-confidence SNPs.

SAMtools mpileup, VCF and coverage files were used to generate the combined nucleotide frequencies among isolates of each positional SNP. The multiple sequence alignment CSV file was converted to FASTA format. The maximum-likelihood (ML) method with a general time-reversible (GTR) model of nucleotide substitution and a gamma-distribution model of rate heterogeneity was selected as the best model, implemented within MEGA version 10.0.5 [30]. A phylogenetic tree was constructed using Randomized Axelerated Maximum Likelihood (RAxML) version 8.2.12 [31] with a consensus tree constructed from 1,000 bootstrap replicates. The phylogenetic tree was visualized using iTOL software [32]. *Mycobacterium chelonae* CCUG 47445 (CP007220.1) was used as the outgroup. Pairwise SNP distance matrices were analyzed using snp-dists version 0.8.2 [33].

### Detection of antimicrobial resistance genes

Antimicrobial resistance genes were identified by conducting mass screening on the assembled MAC genomes using ABRicate software [34] with multiple databases including NCBI, CARD, ARG-ANNOT, ResFinder and MEGARes.

### Detection of genetic mutations associated with drug resistance

The sequences of *rrs*, *rrl*, *rplC*, *rplD*, *gyrA* and *gyrB* genes were extracted from the assembled MAC genome sequences. Mutations in these genes were analyzed by performing multiple sequence alignments against corresponding sequences of the reference genome of *M*. *avium* 104 or *M*. *intracellulare* ATCC 13950 (CP003322.1) using the MUSCLE algorithm tool provided by MEGA.

### Statistical analysis

Statistical analysis was performed using IBM SPSS Statistics for Windows, version 28.0 software (IBM Corp, Armonk, NY, USA). The number of resistant isolates was compared between the two species using the chi-squared or Fisher’s exact test. P-values less than 0.05 were considered statistically significant.

## Results

### Characteristics of the studied isolates

**Fig 1:**
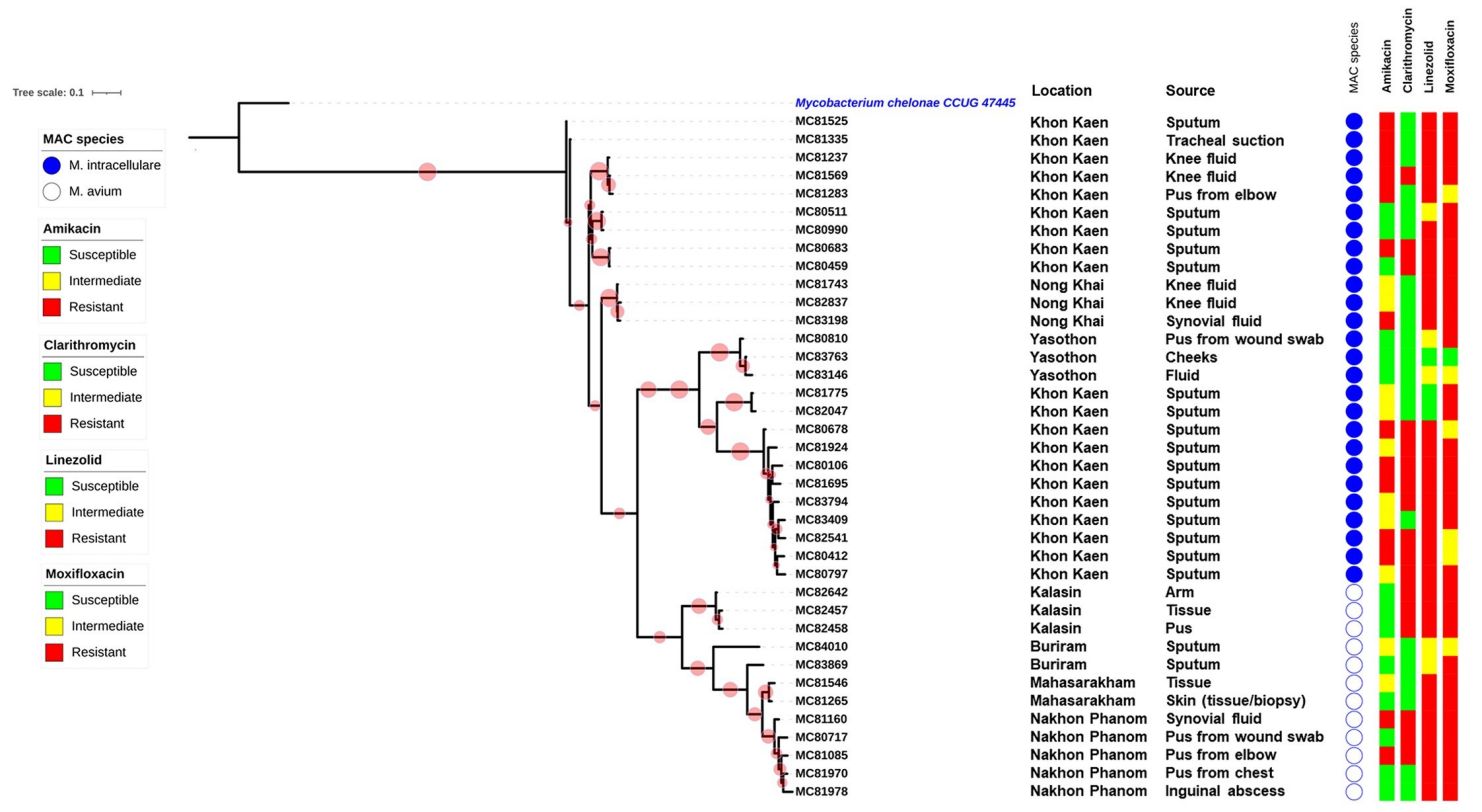
Maximum-Likelihood phylogenetic tree of 38 *Mycobacterium avium* complex clinical isolates. Characteristics of isolates including location of patient and source of specimen, as well as drug-susceptibility profiles for four drugs are provided. All 38 isolates were identified as either *M*. *avium* (indicated by an empty circle) or *M*. *intracellulare* (indicated by a blue circle) based on genome analysis. The phylogenetic tree was generated using iTOL software and a bootstrap consensus tree was inferred from 1,000 replicates. The red circles on the tree refer to bootstrap values, with the size of each circle proportional to its value (the largest red circle indicating a value of 100%). *Mycobacterium chelonae* CCUG 47445 was used as the outgroup.

Thirty-eight clinical isolates of MAC were recovered on LJ medium. Based on LPA analysis, 12 isolates were identified as *M*. *avium* (31.6%) and 26 isolates to *M*. *intracellulare* (68.4%). The majority of the isolates were collected from female patients (63.1%) with an average age of 57 years, ranging from 27 to 91 years. Patients were from many provinces in Northeast Thailand, including Buriram, Kalasin, Khon Kaen, Mahasarakham, Nakhon Phanom, Nong Khai and Yasothon. The characteristics of the MAC isolates are shown in **Fig 1**.

### Phenotypic drug susceptibility of MAC isolates

The drug-susceptibility profiles of all tested MAC isolates are presented in **Table 1 and S1 Table**. The MIC distributions for all drugs were unimodal, except for clarithromycin, ethionamide and rifabutin, which displayed bimodal distributions (**S1 Fig**). The MIC breakpoints for four drugs (amikacin, clarithromycin, linezolid and moxifloxacin) were interpreted according to the approved guidelines established by the CLSI. The most common resistance phenotypes observed were those to linezolid (81.6%), followed by moxifloxacin (78.9%), clarithromycin (44.7%) and amikacin (36.8%). The two MAC species did not differ significantly in the proportion of isolates resistant to each drug.

**Table 1:** Minimum inhibitory concentration (MIC) values for 13 drugs tested against the *Mycobacterium avium* complex clinical isolates.

	Drug^a^ (MIC as μg/mL)												
Total (n = 38)	AMI	CIP	CLA	DOX	EMB	ETH	INH	LZD	MXF	RFB	RIF	STR	SXT
MIC_50_	32	16	8	16	16	20	8	32	8	2	8	64	8/152
MIC_90_	>64	>16	64	>16	>16	>20	>8	>64	>8	8	>8	>64	>8/152
Range	2->64	1->16	0.5->64	16->16	4->16	0.6->20	8->8	8->64	1->8	0.5->8	0.5->8	4->64	2/38->8/152
Resistant (%)	14 (36.8)	NA	17 (44.7)	NA	NA	NA	NA	30 (78.9%)	31 (81.6%)	NA	NA	NA	NA
***M*. *avium* (n = 12)**													
MIC_50_	16	16	4	16	16	20	8	32	8	1	8	32	8/152
MIC_90_	32	>16	64	>16	16	>20	>8	64	>8	8	>8	64	8/152
Range	8->64	16->16	1->64	16->16	4->16	5->20	8->8	16->64	2->8	0.5->8	1->8	16–64	4/76->8/152
Resistant (%)	2 (16.7)	NA	6 (50)	NA	NA	NA	NA	10 (83.3)	11 (91.7)	NA	NA	NA	NA
***M*. *intracellulare* (n = 26)**													
MIC_50_	32	16	8	16	16	5	8	32	4	2	8	64	8/152
MIC_90_	64	16	64	>16	16	>20	>8	64	8	8	>8	64	8/152
Range	2->64	1->16	0.5->64	16->16	8->16	0.6->20	8->8	8->64	1->8	0.5->8	0.5->8	4->64	2/38->8/152
Resistant (%)	12 (46.2)	NA	11 (42.3)	NA	NA	NA	NA	20 (76.9%)	20 (76.9%)	NA	NA	NA	NA
P-value^b^	0.147	NA	0.658	NA	NA	NA	NA	1.000	0.395	NA	NA	NA	NA

^a^ AMI, amikacin; CIP, ciprofloxacin; CLA, clarithromycin; DOX, doxycycline; EMB, ethambutol; ETH, ethionamide; INH, isoniazid; LZD, linezolid; MXF, moxifloxacin; RFB, rifabutin; RIF, rifampicin; STR, streptomycin; SXT, trimethoprim/sulfamethoxazole.

^b^ P-values were calculated using the Chi-squared or Fisher’s exact analysis for comparison the number of resistant isolates between *M*. *avium* and *M*. *intracellulare* groups

NA, not applicable because of the absence of CLSI guidelines for this drug.

Among the *M*. *avium* isolates, 91.7% (11/12) were phenotypically resistant to moxifloxacin, followed by linezolid (83.3%, 10/12), clarithromycin (50.0%, 6/12) and amikacin (16.7%, 2/12). The MIC_50_/MIC_90_ values of the isolates for amikacin, clarithromycin, linezolid and moxifloxacin were 16/32, 4/64, 32/64 and 8/>8 μg/mL, respectively.

Among the *M*. *intracellulare* isolates, the most common resistance phenotypes were those to linezolid and moxifloxacin (76.9%, 20/26), followed by amikacin (46.2%, 12/26) and clarithromycin (42.3%, 11/26). The MIC_50_/MIC_90_ values of the isolates for amikacin, clarithromycin, linezolid and moxifloxacin were 32/64, 8/64, 32/64 and 4/8 μg/mL, respectively.

### Whole-genome sequencing and phylogenetic analysis

A total of 38 MAC isolates were sequenced and their genomes were annotated. The completeness and contamination of the genomes were on average >99% and <3%, respectively, which provides confidence in the results. The average genome size was 5,827,852.8 bp (ranging from 5,311,254 to 6,570,536 bp), and the average GC content was 67.8%. The N50 mean value was 457,039.5 (ranging from 125,728 to 1,338,815) (**S2 Table**).

Among the 38 MAC clinical isolates there was an average pairwise difference of 191 SNPs, ranging from 89 to 415 SNPs. The pairwise differences averaged 243 SNPs for *M*. *avium* isolates (ranging from 115 to 415 SNPs) and 168 SNPs for *M*. *intracellulare* isolates, with a range of 89 to 290 SNPs (**S2 Table**). Based on the phylogenetic tree constructed using SNPs, the MAC isolates were not clonal strains (**Fig 1**).

### Mass screening for antimicrobial resistance genes

Based on ABRicate mass screening, five databases (ARG-ANNOT, CARD, MEGARes, NCBI and ResFinder) were used to identify genes associated with antimicrobial resistance. No such genes were identified in the ARG-ANNOT, NCBI and ResFinder databases. However, three were identified in all isolates using the MEGARes database: drug and biocide MFS efflux pumps (EFPA), multi-drug RND efflux regulator (MTRAD) and RNA-polymerase binding protein (RBPA). The CARD database identified *efpA*, *RbpA* and *rpoB2* as antimicrobial resistance genes (**S3 Table**).

### Mutations in genes associated with phenotypic drug resistance

For amikacin, the *rrs* mutation confers aminoglycoside resistance. A C464T mutation in the *rrs* gene was found in one *M*. *avium* isolate with intermediate resistance. Two mutations (T250A and G1453T) were found in the amikacin non-susceptible (intermediate and resistant) *M*. *intracellulare* isolates (**Table 2 and S4 Table**).

**Table 2:** Drug susceptibility and nucleic acid mutations in *rrs*, *rrl*, *rplC*, *rplD*, *gyrA* and *gyrB* genes in non-susceptible *Mycobacterium avium* and *Mycobacterium intracellulare* clinical isolates.

Drug	Gene	Nucleic acid mutation position found in non-susceptible isolate	
		*Mycobacterium avium* (n = 12)	*Mycobacterium intracellulare* (n = 26)
Amikacin	*rrs* ^*a*^	C464T	T250A, G1453T
Clarithromycin	*rrl* ^*a*^	T321C, G1180A, **A2058G**, **A2059C**, **A2059G**, T2131G	C304T, C1176G, G1240A, G1681A, A2058G, C2209G, G2215C, C2236T, C2402T, C2404T, C2825T, C2840T
Linezolid	*rplC*	G6A, C7A, T9A, G12A, C21G, A48G, A64C, C69T, G84C, G96C, C114G, C120T, G123C, A131G, G132C, G147C, G159C, G177C, G186C, G198C, C204T, G216C, A223G, C225G, **G237C**, T240C, C250T, C261A, C261G, C264T, G267C, C270G, G273C, G276C, A282G, C286G, A319G, A328G, G329C, C345G, A348C, C366T, G387C, C399T, T402C, C420T, G438C, G441C, G444C, C450G, **C459T**, C468T, C471G, A493C, G499T, C501G, C513T, A515G, C519T, G522C, G528C, C577T, C588T, C594T, C609T, C618G, A619G, A639G, A653G	G123C, C240T, G273A, G273C, G279T, C387G
	*rplD*	C9G, C13A, G21C, G25A, G32A, T48C, G51C, C58T, A68T, C78T, C81G, C84G, C90T, G111C, G114C, G120C, G126A, G132C, G135A, G147C, G156C, C165T, C177T, C186G, C222G, T225A, G237C, C240T, T243C, C258T, T261C, G265A, G267C, C276G, C279G, G285C, C291T, G306C, C309G, C327G, C331T, G342A, C357G, G366C, G387A, C388A, G390C, G393T, T394G, C396G, C399T, G402A, C405G, G408T, C414G, G419A, A424C,A425G, G429T, C442T, G450C, G460C, G468C, G471C, **A489G**, G490A, G516C, T522C, A529C, C531G, C532T, G535A, T537C, C558T, G571T, C591T, C594T, G603C, C604A, C604G, G605A, C621G, A623C, A625G, C626G, A631G, T645A, T646A, C651G, G653A, A654G	C39T, G126A, C156G, G159A, G165A, C213T, G243C, G264C, C282T, C285G, C309T, T349C, T357G, G360A, G396C, G420A, G426T, C432A, **G443A**, C450T, C451T, A457G, G507A, C558G, C561T, C606T, A607T, G608C
Moxifloxacin	*gyrA* ^*b*^	G225T, C231G, C234G, A237G, C246T, G258C, C270G, G291A, G294C, C300G, G303A, T321C, T325C, G336T, T345C, T348C	G219C, A222T, G225C, G225T, C231G, C234G, T246C, C252T, A258G, C261T, C267T, G268T, C270T, C285A, C285G, C286T, G291A, G306T, C324T, G336C, C348T, G354C
	*gyrB* ^*b*^	C1335T, G1341C, C1347T, G1350A, G1359C, T1363C, C1374G, T1380C, C1383T, C1389G, C1392T, C1395T, G1401C, A1404G, C1407T, G1413C, C1431G, T1437G, C1473G, C1489T, G1506C, G1506T, G1536C, G1536T, T1539C, C1542T, C1545T, G1548A, A1558G, C1569T, C1572T	C1332T, G1350A, C1353G, G1356A, G1365C, T1368C, G1374C, T1380C, G1386A, C1389A, G1401C, G1404A, C1407T, A1431C, G1470A, C1473G, G1485C, A1548G, A1558G, C1559G

^*a*^ *Escherichia coli* numbering

^*b*^ Nucleotide sequences from 217 to 351 for *gryA* and from 1330 to 1581 for *gyrB* in the *Mycobacterium tuberculosis* numbering [13]

Non-susceptible isolates, intermediate- and resistant drug isolates

Mutations in bold are concordant with published studies.

For clarithromycin, mutations in *rrl* confer macrolide resistance. Only clarithromycin-resistant *M*. *avium* isolates had *rrl* mutations at positions T321C, G1180A, A2058G, A2059C, A2059G and T2131G. For *M*. *intracellulare*, several mutations in the *rrl* gene were found in the non-susceptible isolates, including C304T, C1176G, G1240A, G1681A, A2058G, C2209G, G2215C, C2236T, C2402T, C2404T, C2825T and C2840T (**Table 2 and S4 Table**).

For linezolid, the *rplC* and *rplD* genes were investigated for resistance. This study did not include any *M*. *avium* isolates susceptible to linezolid, so a comparison between mutations in susceptible and non-susceptible isolates could not be made. However, several mutations were found in the *rplC* gene of non-susceptible *M*. *avium* isolates. Among *M*. *intracellulare* isolates, six mutations in the *rplC* gene were found, all of which were synonymous in linezolid non-susceptible isolates. In addition, we found some mutations in the *rplD* gene, two of which were non-synonymous (Arg148Lys and Thr153Ala) in linezolid non-susceptible *M*. *intracellulare* isolates (**Tables 2 and 3 and S4 and S5 Tables**).

**Table 3:** Drug susceptibility and amino acid substitutions in 50S ribosomal protein L3, 50S ribosomal protein L4, GyrA and GyrB proteins in non-susceptible *Mycobacterium avium* and *Mycobacterium intracellulare* clinical isolates.

Drug	Protein	Amino acid substitutions position found in non-susceptible isolate	
		*Mycobacterium avium* (n = 12)	*Mycobacterium intracellulare* (n = 26)
Linezolid	50S ribosomal protein L3	Q44R, I75V, D87E, D90E, Q96E, T107A, S110A, A167S, N172S, M207V	Not found
	50S ribosomal protein L4	L3V, L5I, A9T, G11D, E23V, V89I, L130I, S132A, S140N, K142R, E150D, V154L, A164T, I177L, A179T, A191S, R202G, R202N, N208T, T209G, S217T	R148K, T153A
Moxifloxacin	GyrA^*a*^	Not found	A91S
	GyrB^*a*^	T521A	T521A, T521S

^*a*^ Peptide sequences from codon 74 to 113 for GyrA and from codon 461 to 499 for GyrB in the *Mycobacterium tuberculosis* numbering [13]

Non-susceptible isolates, intermediate- and resistant-drug isolates

Not found, no amino acid substitutions relative to corresponding sequences of the reference genome of *M*. *avium* 104 or *M*. *intracellulare* ATCC 13950.

For moxifloxacin, mutations in the quinolone resistance-determining regions (QRDRs) of *gyrA* and *gyrB* were identified as being associated with fluoroquinolone resistance. Several mutations were found in *gyrA* and *gyrB* genes of *M*. *avium* isolates. However, due to the absence of moxifloxacin-susceptible *M*. *avium* isolates, mutations associated with moxifloxacin resistance could not be determined. In *M*. *intracellulare*, several mutations of the *gyrA* gene were found, all of which were synonymous, except for one causing the amino acid substitution Ala91Ser. Moreover, two amino acid substitutions (Thr521Ala and Thr521Ser) of the GyrB protein were found only in non-susceptible moxifloxacin isolates (**Tables 2 and 3 and S4 and S5 Tables**).

## Discussion

This study reports the MIC values (determined by broth microdilution) for 13 representative drugs in 38 MAC clinical isolates belonging to *M*. *avium* and *M*. *intracellulare*. The CLSI guidelines were used to investigate the MIC distribution for four drugs (amikacin, clarithromycin, linezolid and moxifloxacin) and to define drug resistance. However, breakpoints for defining resistance against the other drugs have not yet been established [8]. We found variations in MIC values similar to those previously reported [35]. The majority of isolates (81.6%) exhibited resistance to moxifloxacin, followed by linezolid (78.9%), clarithromycin (44.7%) and amikacin (36.8%). The MIC distribution of all drugs was broad (≥ 5 dilutions), except for isoniazid, doxycycline, ethambutol and trimethoprim/sulfamethoxazole, which all had a narrow range with high concentrations. The proportion of isolates resistant to each drug was not statistically significantly different between *M*. *avium* and *M*. *intracellulare*. Nevertheless, the rates of resistance to moxifloxacin, linezolid, clarithromycin and amikacin in this study appear to be higher than those reported in some previous studies [12, 13, 36]. Most of the isolates we tested showed similar susceptibility levels for most drugs, as demonstrated by the unimodal MIC distributions. However, bimodal distributions were observed for clarithromycin, ethionamide and rifabutin, which may suggest the presence of two distinct mycobacterial subpopulations with varying susceptibility levels or different mechanisms of resistance.

Amikacin is an important parenteral drug for treating NTM, especially MAC and *M*. *abscessus* [37]. We detected amikacin resistance in 16.7% and 46.2% of *M*. *avium* and *M*. *intracellulare* isolates, respectively. A previous study found that most *M*. *avium* isolates were phenotypically susceptible to amikacin (85.9%), while no isolate was resistant [12]. The MIC_50_/MIC_90_ values for amikacin were 16/32 and 32/64 μg/mL for *M*. *avium* and *M*. *intracellulare* isolates, respectively. To the best of our knowledge, the mechanism of high-level resistance in MAC isolates involves mutations in the *rrs* gene. A1408G, C1409T and G1419T are the most common mutations detected in this gene in kanamycin-resistant *M*. *tuberculosis* [38]. A recent study identified novel mutations at G1491T and G1491C, and two additional mutations at C1496T and T1498A, in amikacin-resistant mycobacteria isolates [11]. Among the mutations found in *M*. *intracellulare* isolates, two (T250A and G1453T) were only in amikacin-intermediate and -resistant isolates. Therefore, we suggest that those mutations might be involved in amikacin resistance. Although these mutations were not present in all of our non-susceptible isolates, other mechanisms may contribute to aminoglycoside resistance. Previous literature suggests that aminoglycoside acetyltransferase and the drug efflux pump are associated with aminoglycoside resistance [39].

Clarithromycin is a macrolide drug and the key therapeutic agent for NTM diseases [20] The CLSI guidelines recommend that susceptibility testing is done for this drug [8]. A study by Wetzstein et al. [12] reported a low prevalence (≈3%) of MAC isolates resistant to clarithromycin. Similarly, in Maurer et al. [40] and Litvinov et al. [41], macrolide resistance was also rare. On the other hand, nearly half of the MAC isolates in our study (44.7%; 17 of the 38 isolates) were resistant to clarithromycin. It has been suggested that clarithromycin resistance is increasing in NTM. Studies of clarithromycin resistance in MAC isolates have identified resistance-associated mutations (A2058 and A2059) in the 23S rRNA gene (*rrl*) [42–45]. Our results agree with these findings, with the *rrl* mutation A2058G found in MAC isolates with clarithromycin resistance. Additional mutations A2059G and A2059C were also found only in clarithromycin-resistant *M*. *avium* isolates. Thus, *rrl* mutations (A2058G, A2059G and A2059C) are potentially associated with clarithromycin resistance.

Recent clinical guidelines recommend linezolid as a treatment option for drug-resistant TB and NTM infections [46, 47]. However, studies of linezolid susceptibility in NTM have reported different resistance rates according to species [48–50]. Our findings showed that most MAC clinical isolates were highly resistant to linezolid (78.9%), including 83.3% of *M*. *avium* and 76.9% of *M*. *intracellulare* isolates. These results are consistent with a previous study in Korea [14], which found 52.7% and 50% resistance to linezolid among *M*. *avium* and *M*. *intracellulare* isolates, respectively. The *rplC* and *rplD* genes encode for the 50S ribosomal proteins L3 and L4, respectively, and mutations in these genes have been associated with resistance to linezolid in Gram-positive bacteria [51]. In our study, several mutations were found in both *M*. *avium* and *M*. *intracellulare* isolates. In *M*. *avium*, some mutations, such as G237C and C459T in *rplC* and A489G in *rplD*, were concordant with previous studies [14] on linezolid resistance. In *M*. *intracellulare*, six mutations (all synonymous) were found in the *rplC* gene only in isolates non-susceptible to linezolid. In addition, several mutations were found in the *rplD* gene. One of these mutations (G443A) was found in non-susceptible *M*. *intracellulare* isolates, in agreement with a previous report [14]. Another study reported that C366G and T534G mutations in the *rplD* gene were associated with resistance to linezolid [14], but we found these mutations in both susceptible and resistant isolates. Two additional amino acid substitutions, Arg148Lys (R148K) and Thr153Ala (T153A), were found only in our linezolid-resistant isolates, which could be associated with phenotypic resistance to this drug. However, we suggest that the various observed mutations might be affected by high genetic diversity in MAC isolates. Overall, mutations in the *rplC* and *rplD* genes have been associated with resistance to linezolid in MAC.

As per the ATS/IDSA guidelines, fluoroquinolones such as moxifloxacin and levofloxacin are the recommended drugs for the treatment of macrolide-resistant MAC and pulmonary *M*. *abscessus* disease [20, 46, 52]. However, it is important to note that resistance to fluoroquinolones has been observed, and the use of these drugs must be carefully monitored by clinicians [13, 36]. Mutations in DNA gyrase subunits, encoded by the *gyrA* and *gyrB* genes in the QRDRs, have been identified as associated with fluoroquinolone resistance [53]. Some mutations in *gyrA* and *gyrB* in *M*. *avium* might be associated with this resistance [54]. In contrast, the largest reported study showed that no amino acid substitution in GyrA or GyrB was associated with moxifloxacin resistance in MAC, and they suggested that other mechanisms, such as efflux pumps, are involved in moxifloxacin resistance [13]. Another study reported that mutations in the QRDRs of *gyrA* and *gyrB* were not found among the moxifloxacin-resistant *M*. *avium* and *M*. *intracellulare* isolates [36]. In our study, several mutations in *gyrA* and *gyrB* genes in *M*. *avium* were observed and one amino acid substitution was identified, Thr521Ala (T521A) in GyrA. Some of these mutations were consistently present among moxifloxacin non-susceptible *M*. *intracellulare* isolates. Interestingly, we found that the GyrB substitutions Thr521Ala (T521A) and Thr521Ser (T521S) were present only in non-susceptible moxifloxacin isolates, similar to *M*. *avium*. Our finding suggests that the GyrB substitutions Thr521Ala (T521A) or Thr521Ser (T521S) were present only in *M*. *avium* and *M*. *intracellulare* isolates that were not susceptible to moxifloxacin, which might contribute to moxifloxacin resistance. However, such association should be further investigated with a larger sample size. Also, further studies are needed to better understand the molecular mechanisms of drug resistance in MAC isolates.

The genome of MAC was relatively large, consisting of approximately 5.8 Mbp. Studies of the MAC genome has identified numerous genes and pathways involved in virulence and drug resistance as well as variation in gene content and organization among different strains, which could contribute to differences in virulence and drug resistance [55, 56]. Understanding the functions of these virulence factors is crucial for the development of effective therapies against mycobacterial infections. Our investigation of the MAC genome has yielded invaluable insights into the biology and pathogenesis of these bacteria and has the potential to lead to the development of new treatments for MAC infections.

Limitations of our study should be noted. First, the number of MAC isolates tested was limited. Future studies with larger sample sizes are required to draw more meaningful conclusions. Furthermore, several mutations observed in our tested isolates might have simply reflected the high genetic diversity of MAC. While some mutations have been reported previously, supporting the view that these are associated with drug resistance in MAC, further validation is required for certain novel mutations or amino acid substitutions that we found. Additional studies that evaluate the mutations found in non-susceptible isolates in-depth and investigate those associated with phenotypic resistance are warranted. The mutation databases associated with each of the antibiotics or MAC are still very limited. Thus, we could not properly compare the performance of genotypic drug-susceptibility determination with phenotypic drug-susceptibility testing. Instead, we analyzed mutations in genes known from other mycobacteria to be associated with phenotypic drug resistance to get an insight into the drug resistance mechanism of MAC.

In conclusion, our study evaluated the drug susceptibility of MAC isolates and revealed high rates of resistance to moxifloxacin, followed by linezolid, clarithromycin and amikacin. High resistance rates might be a consequence of the misuse of antibiotics in our region [57]. Specifically, we found an *rrs* mutation at C464T for amikacin intermediate resistance in *M*. *avium*, and two mutations (T250A and G1453T) in amikacin non-susceptible *M*. *intracellulare*. Mutations in *rrl* at A2058G, A2059C and A2059G were potentially linked to clarithromycin resistance. Non-susceptibility to linezolid was associated with mutations in *rplC* at G237C and C459T, as well as two *rplD* mutations at G443A and A489G. We also identified the GyrB substitution Thr521Ala (T521A) in moxifloxacin non-susceptible isolates, which may contribute to resistance against that drug. Our findings provide insights into the specific mutations associated with drug resistance in MAC clinical isolates. These results emphasize the importance of using both phenotypic and genotypic susceptibility testing to assist clinicians in selecting effective drugs for the treatment of MAC infections. Due the high resistance rate we found to the antibiotics used for treatment of MAC infection, especially moxifloxacin and linezolid, new treatment strategies and adjustment of antibiotic options are needed.

## Supporting information

S1 Table Minimum inhibitory concentration (MIC) values of 13 drugs determined for 38 *Mycobacterium avium* complex clinical isolates. (DOCX)

S2 Table General statistics and features of the whole-genome sequences of 38 *Mycobacterium avium* complex clinical isolates. (XLSX)

S3 Table Genes associated with antimicrobial resistance detected using different five databases by ABRicate mass screening of 38 *Mycobacterium avium* complex clinical isolates. (XLSX)

S4 Table Drug susceptibility and nucleic acid mutations in *rrs*, *rrl*, *rplC*, *rplD*, *gyrA* and *gyrB* genes in 38 *Mycobacterium avium* complex clinical isolates. (XLSX)

S5 Table Drug susceptibility and amino acid substitutions in 50S ribosomal protein L3, 50S ribosomal protein L4, GyrA and GyrB proteins in 38 *Mycobacterium avium* complex clinical isolates. (XLSX)

S1 Fig Minimum inhibitory concentration (MIC) distributions of 13 drugs tested against 38 clinical isolates of *Mycobacterium avium* complex species. (TIF)
