# Supplementary material for: Phenotypic drug-susceptibility profiles and genetic analysis based on whole-genome sequencing of Mycobacterium avium complex isolates in Thailand
Source: PLoS One. 2023 Nov 22;18(11):e0294677. doi: 10.1371/journal.pone.0294677 (PMC10664917; doi:10.1371/journal.pone.0294677)
Supplement: S1 Table — (DOCX) [file pone.0294677.s001.docx]

**S1 Table.** Minimum inhibitory concentration (MIC) values of 13 drugs determined for 38 *Mycobacterium avium* complex clinical isolates.

| **Species** | **Isolate** | **Drug^a^ (MIC as µg/mL)** | | | | | | | | | | | | |
| --- | --- | --- | --- | --- | --- | --- | --- | --- | --- | --- | --- | --- | --- | --- |
|  |  | **AMI** | **CIP** | **CLA** | **DOX** | **EMB** | **ETH** | **INH** | **LZD** | **MXF** | **RFB** | **RIF** | **STR** | **SXT** |
| **Breakpoint value (µg/mL)^b^** | | ≥64 | - | ≥32 | - | - | - | - | ≥32 | ≥4 | - | - | - | - |
| Total  (n=38) | MIC_50_ | 32 | 16 | 8 | 16 | 16 | 20 | 8 | 32 | 8 | 2 | 8 | 64 | 8/152 |
|  | MIC_90_ | >64 | >16 | 64 | >16 | >16 | >20 | >8 | >64 | >8 | 8 | >8 | >64 | >8/152 |
|  | Range | 2->64 | 1->16 | 0.5->64 | 16->16 | 4->16 | 0.6->20 | 8->8 | 8->64 | 1->8 | 0.5->8 | 0.5->8 | 4->64 | 2/38->8/152 |
|  | %Resistance | 36.8 | NA | 44.7 | NA | NA | NA | NA | 78.9 | 81.6 | NA | NA | NA | NA |
| *M. avium*  (n=12) | MC80717 | 8 | 16 | 64 | 16 | 16 | 20 | 8 | 32 | 8 | 1 | 8 | 64 | 8/152 |
|  | MC81085 | 64 | >16 | >64 | >16 | >16 | >20 | >8 | >64 | >8 | >8 | >8 | 64 | >8/152 |
|  | MC81160 | >64 | >16 | >64 | >16 | >16 | >20 | >8 | >64 | >8 | 8 | >8 | 32 | >8/152 |
|  | MC81265 | 16 | >16 | 8 | >16 | 4 | 20 | >8 | 32 | >8 | 1 | >8 | 32 | 4/76 |
|  | MC81546 | 32 | >16 | 1 | >16 | 8 | >20 | >8 | 32 | >8 | 2 | >8 | 32 | 4/76 |
|  | MC81970 | 16 | 16 | 4 | 16 | 16 | 20 | 8 | 32 | 8 | 4 | 8 | 64 | 8/152 |
|  | MC81978 | 16 | 16 | 4 | 16 | 16 | 10 | 8 | 64 | 8 | 8 | 8 | 32 | 8/152 |
|  | MC82457 | 8 | 16 | 64 | 16 | 16 | 5 | 8 | 32 | 8 | 0.5 | 8 | 32 | 8/152 |
|  | MC82458 | 8 | 16 | 64 | 16 | 16 | 5 | 8 | 32 | 8 | 0.5 | 8 | 32 | 8/152 |
|  | MC82642 | 8 | 16 | 64 | 16 | 16 | 20 | 8 | 32 | 8 | 1 | 8 | 16 | 8/152 |
|  | MC83869 | 16 | 16 | 1 | 16 | 16 | 20 | 8 | 16 | 8 | 2 | 8 | 32 | 8/152 |
|  | MC84010 | 32 | 16 | 2 | 16 | 8 | 10 | 8 | 16 | 2 | 0.5 | 1 | 64 | 8/152 |
| *M. intracellulare*  (n=26) | MC80106 | 64 | 16 | 64 | 16 | 16 | 2.5 | 8 | 64 | 8 | 8 | 8 | 64 | 8/152 |
|  | MC80412 | 64 | 16 | 64 | 16 | 16 | 1.2 | 8 | 32 | 2 | 8 | 8 | 64 | 8/152 |
|  | MC80459 | 8 | 16 | 64 | 16 | 16 | 20 | 8 | 32 | 8 | 2 | 8 | 64 | 8/152 |
|  | MC80511 | 16 | 16 | 1 | 16 | 16 | 20 | 8 | 16 | 8 | 2 | 8 | 32 | 8/152 |
|  | MC80678 | 64 | 16 | 32 | 16 | 8 | 1.2 | 8 | 32 | 2 | 8 | 8 | 64 | 8/152 |
|  | MC80683 | 64 | 16 | 64 | 16 | 16 | 20 | 8 | 64 | 8 | 8 | 8 | 64 | 8/152 |
|  | MC80797 | 32 | 16 | 64 | 16 | 16 | 5 | 8 | 32 | 8 | 8 | 8 | 64 | 8/152 |
|  | MC80810 | 8 | 16 | 1 | 16 | 16 | 1.2 | 8 | 16 | 4 | 0.5 | 4 | 16 | 8/152 |
|  | MC80990 | 16 | 16 | 2 | 16 | 16 | 10 | 8 | 64 | 8 | 2 | 8 | 64 | 8/152 |
|  | MC81237 | >64 | >16 | 4 | >16 | >16 | >20 | >8 | >64 | 4 | 4 | >8 | >64 | >8/152 |
|  | MC81283 | 64 | 16 | 4 | >16 | >16 | >20 | >8 | 64 | 2 | 1 | >8 | 64 | 4/76 |
|  | MC81335 | >64 | >16 | 4 | >16 | >16 | >20 | >8 | >64 | >8 | 8 | >8 | >64 | >8/152 |
|  | MC81525 | 64 | >16 | 4 | >16 | 8 | >20 | >8 | 64 | 8 | 4 | >8 | >64 | 4/76 |
|  | MC81569 | >64 | >16 | >64 | >16 | >16 | >20 | >8 | 64 | >8 | >8 | >8 | >64 | >8/152 |
|  | MC81695 | 64 | 16 | 64 | 16 | 16 | 20 | 8 | 64 | 4 | 8 | 8 | 64 | 8/152 |
|  | MC81743 | 32 | 16 | 2 | 16 | 16 | 20 | 8 | 32 | 4 | 0.5 | 8 | 64 | 8/152 |
|  | MC81775 | 32 | 1 | 8 | 16 | 8 | 0.6 | 8 | 8 | 4 | 0.5 | 2 | 32 | 8/152 |
|  | MC81924 | 32 | 16 | 64 | 16 | 16 | 1.2 | 8 | 32 | 4 | 8 | 8 | 64 | 8/152 |
|  | MC82047 | 32 | 1 | 8 | 16 | 8 | 0.6 | 8 | 8 | 4 | 0.5 | 2 | 32 | 8/152 |
|  | MC82541 | 64 | 16 | 64 | 16 | 16 | 5 | 8 | 64 | 2 | 8 | 8 | 64 | 8/152 |
|  | MC82837 | 32 | 16 | 2 | 16 | 8 | 20 | 8 | 32 | 4 | 0.5 | 8 | 64 | 4/76 |
|  | MC83146 | 8 | 16 | 1 | 16 | 16 | 1.2 | 8 | 16 | 2 | 2 | 0.5 | 16 | >8/152 |
|  | MC83198 | 64 | 16 | 2 | 16 | 8 | 20 | 8 | 64 | 4 | 0.5 | 8 | 64 | 2/38 |
|  | MC83409 | 32 | 16 | 8 | 16 | 8 | 1.2 | 8 | 32 | 4 | 8 | 8 | 64 | 8/152 |
|  | MC83763 | 2 | 4 | 0.5 | 16 | 16 | 1.2 | 8 | 8 | 1 | 0.5 | 0.5 | 4 | 8/152 |
|  | MC83794 | 32 | 16 | 64 | 16 | 16 | 20 | 8 | 64 | 4 | 8 | 8 | 64 | 8/152 |

^a^ AMI, amikacin; CIP, ciprofloxacin; CLA, clarithromycin; DOX, doxycycline; EMB, ethambutol; ETH, ethionamide; INH, isoniazid; LZD, linezolid; MXF, moxifloxacin; RFB, rifabutin; RIF, rifampicin; STR, streptomycin; SXT, trimethoprim/sulfamethoxazole.

^b^ Breakpoint values were set by compiling data according to the CLSI.

Gray-shaded boxes show strains with a MIC value equal to or higher than the breakpoint values.

-, break point not established.

NA, not applicable.
