# Supplementary figures and images for: Phenotypic drug-susceptibility profiles and genetic analysis based on whole-genome sequencing of Mycobacterium avium complex isolates in Thailand
Source: PLoS One. 2023 Nov 22;18(11):e0294677. doi: 10.1371/journal.pone.0294677 (PMC10664917; doi:10.1371/journal.pone.0294677)

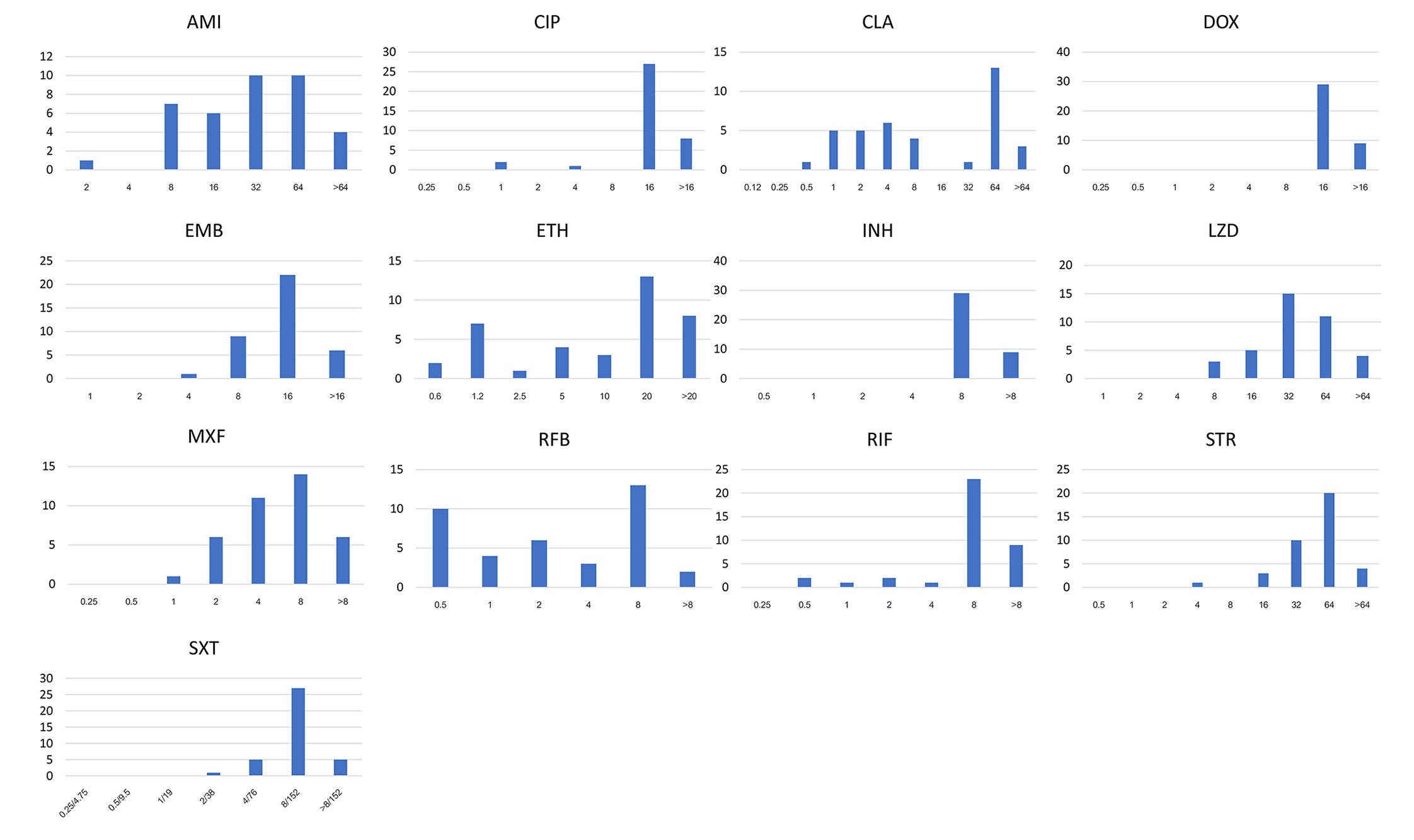

Supplement: S1 Fig — (TIF) [file pone.0294677.s006.tif]
